# Reading specific memories from human neurons before and after sleep

**DOI:** 10.1101/2025.07.01.662486

**Published:** 2025-08-12

**Authors:** Yuanyi Ding, Soraya L. S. Dunn, John J. Sakon, Chenda Duan, Yipeng Zhang, Joel I. Berger, Ariane E. Rhone, Kirill V. Nourski, Hiroto Kawasaki, Matthew A. Howard, Vwani P. Roychowdhury, Itzhak Fried

**Affiliations:** 1 Electrical and Computer Engineering Department, UCLA, Los Angeles, CA, USA.; 2 Department of Neurosurgery, UCLA Health, Los Angeles, CA, USA.; 3 Department of Neurosurgery, Iowa Carver College of Medicine, Iowa City, IA, USA.

**Keywords:** episodic memory, brain decoding, deep learning, transformer, single neuron, intracranial, concept cells, hippocampus, medial temporal lobe, frontal cortex

## Abstract

The ability to retrieve a single episode encountered just once is a hallmark of human intelligence and episodic memory[1]. Yet, decoding a specific memory from neuronal activity in the human brain remains a formidable challenge. Here, we develop a transformer neural network model[2, 3] trained on neuronal spikes from intracranial microelectrodes recorded during a single viewing of an audiovisual episode. Combining spikes throughout the brain via cross-channel attention[4], capable of discovering neural patterns spread across brain regions and timescales, individual participant models predict memory retrieval of specific concepts such as persons or places. Brain regions differentially contribute to memory decoding before and after sleep. Models trained using only medial temporal lobe (MTL) spikes significantly decode concepts before but not after sleep, while models trained using only frontal cortex (FC) spikes decode concepts after but not before sleep. These findings suggest a system-wide distribution of information across neural populations that transforms over wake/sleep cycles[5]. Such decoding of internally generated memories suggests a path towards brain-computer interfaces to treat episodic memory disorders through enhancement or muting of specific memories.

Reading internal cognitive states of the human mind from neural activity, such as specific episodic memories, remains a major challenge for contemporary neuroscience. Emerging approaches involve training deep learning neural network models on electrophysiological activity recorded while participants engage with external stimuli and subsequently uncovering the stimuli from held-out neural data, such as decoding objects in an image[6, 7], speech[8, 9], heard music[10, 11], or characters in a video[12–14]. Methods that decode internal cognitive states[15], however, require many presentations of static images [16, 17] or words[18] to form a training set and typically pool neural data across several participants[13, 18]. A neural mechanism to uncover internal thoughts could use concept cells, neurons that selectively respond to concepts such as persons or places regardless of their sensory input[19], and internally reactivate prior to free recall[20]. Indeed, selective responses from concept neurons can reliably predict memory retrieval of a concept[20, 21] and decode visual inputs using linear methods[16]. Given the sparse coding by MTL concept neurons, however, the ability to identify and potentially target neurons selective to a given concept remains limited[22]for new experiences[13, 23]. Optogenetic methods that selectively target cells, such as engram cells[24], remain unavailable in humans. Further, models using single neuron inputs typically require laborious manual curation after data collection[25], restricting the ability to decode from previously-trained models in real-time.

In sum, decoding of specific memories from one-shot experience based on neural responses remains infeasible with current methods. However, the brain successfully assimilates new experience into durable memory traces extending from the hippocampal-entorhinal system to the neocortex[26]. This distributive process becomes prominent during slow-wave sleep, where the bulk of memory consolidation is thought to occur[5, 27]. We ask, then, if the distribution of episodic information throughout the brain supports a decoding model of specific memories during awake recall, and if so, how the representation and resultant decoding might change following sleep.

To determine if we can identify retrieval of concepts encoded during a single episodic experience, we asked 10 participants undergoing clinical monitoring with intracranial electrodes for intractable epilepsy[28] to watch a single 42-minute episode (from the TV series “24”) and subsequently: 1) perform five minutes verbal free recall of episode content from memory, 2) distinguish 75 “old” video clips (0.5–3.0 s) of the watched episode from 75 “new” clips of an unseen episode in the same series, and 3) verbally answer six cued recall questions for 45 seconds each (Fig. 1a). As we anticipate neural representations to change across initial encoding, subsequent recall, and recall after sleep[5], we repeated the memory tests following overnight sleep. The tests indicate participants encoded the episode well (Fig. 1c), correctly answering 81.0±7.0% of old/new recognition memory questions with all participants achieving a d-prime> 1.0 (Fig. 1f, top; d-prime=1.94 ± 0.54).

As participants performed the tasks we recorded neural activity from microwires (69.6 ± 20.0 electrodes across 10 participants) in various brain regions—particularly Medial Temporal Lobe (MTL) and Frontal Cortex (FC)(Fig. 1a–b)—capable of recording single unit activity[28, 29]. We thresholded all signals from microwires in grey matter to extract clusterless voltage spike trains[30] for each electrode (Methods). This method allows rapid, real-time decoding of new data[31, 32], making our models compatible with future BCI devices. We designed a transformer neural network model[2] that computes channel-wise self-attention within each microwire bundle and then combines information across regions via cross-channel attention[4] (Extended Data Fig. 1a; see Methods). Training of each model occurs separately for each participant using the clusterless voltages recorded while participants watched a single presentation of the episode to identify neuronal patterns related to a set of concepts, including persons (e.g., the main character J. Bauer), places (e.g., the White House), and abstract concepts (e.g. sacrifice) present in the episode (Fig 1e). We chose the eight concepts based on the amount of time on-screen (Fig. 1d–e) and the tendency of participants to recall them (Fig. 1d).

## Memory decoding performance

To predict the identity of concepts in out-of-sample data recorded during recall (pooled free and cued recall where available) we inference from each participant’s model trained on viewing period data. This process outputs a model activation between 0 and 1 for each concept independently over the course of each recall period (Fig. 2a), indicating how strongly the neural pattern at each moment during internally generated memory retrieval matches the signature for that concept acquired from model training. For concepts where models successfully identify associated neuronal patterns, we expect increases in model activation prior to vocalizations of that given concept, thereby reflecting memory reinstatement. To assess such reinstatement, we developed an evaluation metric termed memory confidence score (MCS) (Fig. 2b; Methods), which quantifies the model activation prior to vocalization of a given concept vs. the activations for that concept before non-concept vocalizations (Fig. 2a, bottom). To calculate MCS, first we average the area above baseline −4 to 0 seconds before each vocalization of a given concept (*A*_real_, Fig. 2b, left), where the baseline represents mean activation across the whole recall period for that concept (Fig. 2a, dashed line). We then take an equal number of randomly-sampled vocalizations for other words and repeat this process 1500 times to create a surrogate set of mean concept activations over baseline(${\left\{A_{\text{surrogate}}^{\left(k\right)}\right\}}_{k=1}^{1500}$, Fig. 2b, middle). The MCS represents the percentile rank for the real value vs surrogates (Fig. 2b, right; Fig. 2c, bottom). This way, we determine the MCS for a concept not from the absolute value of model activity, but rather by comparing the statistics of the model output for vocalizations of a specific concept against those of surrogate vocalizations. We limit the evaluation of MCS to concepts vocalized at least three times during recall (Fig. 2d).

Across all ten participants, our model shows significant decoding of concepts during presleep recall with pooled MCS significantly above chance (*Z* = 3.26, *p* = 0.0011, *D* = 0.470; Wilcoxon signed-rank test and one-sample Cohen’s D for all MCS vs. 50% comparisons; Fig. 2d). We show several examples of significantly decoded concepts for single participants in Fig. 2c. In addition, our model significantly decodes the population of concepts during postsleep memory tests (*Z* = 3.46, *p* = 0.0005, *D* = 0.516), indicating robust memory decoding performance. Do we achieve optimal decoding just prior to vocalization, as expected if our model decodes memories? Running identical model inference and MCS metrics as in our main results but on stepwise windows before or after the −4 to 0 s window shows decreasing decoding in both directions (Fig. 3c, left), suggesting that our method unveils internal generation of memories reinstated just prior to vocalization.

We tested several alternative models to confirm the effectiveness of our deep learning decoding approach. First, we fail to see significant decoding using logistic regression (presleep: *Z* = 0.743, *p* = 0.4572, *D* = 0.099, postsleep: *Z* = 0.878, *p* = 0.3800, *D* = 0.121; Extended Data Fig. 2a), suggesting our deep learning model takes advantage of non-linear and spatiotemporal neural population interactions[13, 33]. Second, we attempt a similar decoding framework using broadband oscillatory features in macrowire iEEG data[34], which covers more brain regions but at worse spatial resolution, and decoding performance fails to rise above chance level for either presleep (*Z* = −0.108, *p* = 0.9137, *D* = −0.019) or postsleep (*Z* = 0.573, *p* = 0.5667, *D* = 0.090; Extended Data Fig. 2b). Finally, to ensure our model does not reflect coincident overfitting, we ran 100 surrogate models after circularly shifting microwire channel inputs by a random amount of time in the movie viewing period for each model (Methods). For both pre- and postsleep, all surrogate models show lower median MCS than the correctly-aligned data (presleep: mean *Z* = −0.138, mean *D* = −0.019; postsleep: mean *Z* = 0.174, mean *D* = 0.023; Extended Data Fig. 3).

## MTL and FC differentially contribute to memory decoding before and after sleep

**Fig. 1:**
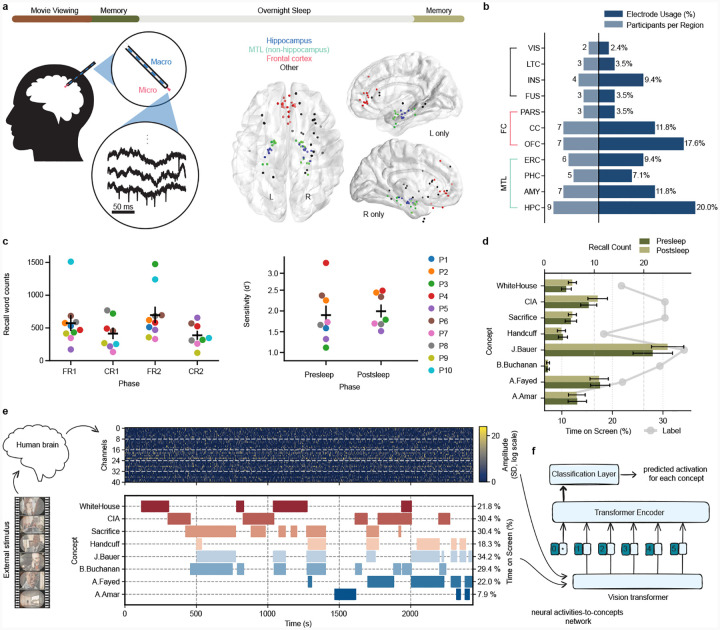
Methods and behavioral results. **a**, Top: Participants watched an episode of the show 24 followed by several memory tests. After sleep, they completed the same set of memory tests the next morning. Left: Throughout the entire experiment, we recorded brain activity using Behnke-Fried depth electrodes with microwires protruding from the macroelectrode into medial brain structures. Center: Localization of microwire recording sites across all participants shown on a 3D axial reconstruction. Right: sagittal views of left hemisphere and right hemisphere electrodes across all participants. **b**, Microwire distribution across brain regions. Bars extending to the right indicate the proportion of electrodes in each region, while bars extending to the left indicate the number of unique participants with electrodes in each region. Abbreviations: HPC (hippocampus), AMY (amygdala), PHC (parahippocampal cortex), ERC (entorhinal cortex), OFC (orbitofrontal cortex), CC (cingulate cortex), FUS (fusiform gyrus), INS (insular cortex), LTC (lateral temporal cortex), PARS (pars orbitalis), VIS (visual cortex). **c**, Total words spoken (left) during free recall (FR) and cued recall (CR) before and after sleep (1 vs. 2) shows that participants reliably recall far more than target concepts. Recognition memory sensitivity (d-prime, right), which assesses true positive vs. false positive accuracy of old/new clips for each participant, confirms reliable memory for movie content before and after sleep. **d**, Average number of vocalized recalls for each concept collected during the presleep and postsleep recall tasks (bars) contrasted with the %time the concept was shown onscreen in the episode (lines). Error bars (black) reflect standard error (SE) across 10 participants. **e**, (Top): example raster plot of positive-polarity spike amplitudes across the movie episode. Gray dashed lines separate bundles of eight microwires. The input matrix has shape 2 × *channels* × *bins*, with the first dimension indexing spike polarity (see Methods). (Bottom): Occurrence of concepts during episode. **f**, Schematic of the encoding model. We trained a vision transformer to learn the mapping between neural activity and the presence of semantic concepts shown in the external stimulus (TV episode). The model aims to identify consistent neural representations associated with each concept across different moments in the movie.

**Fig. 2:**
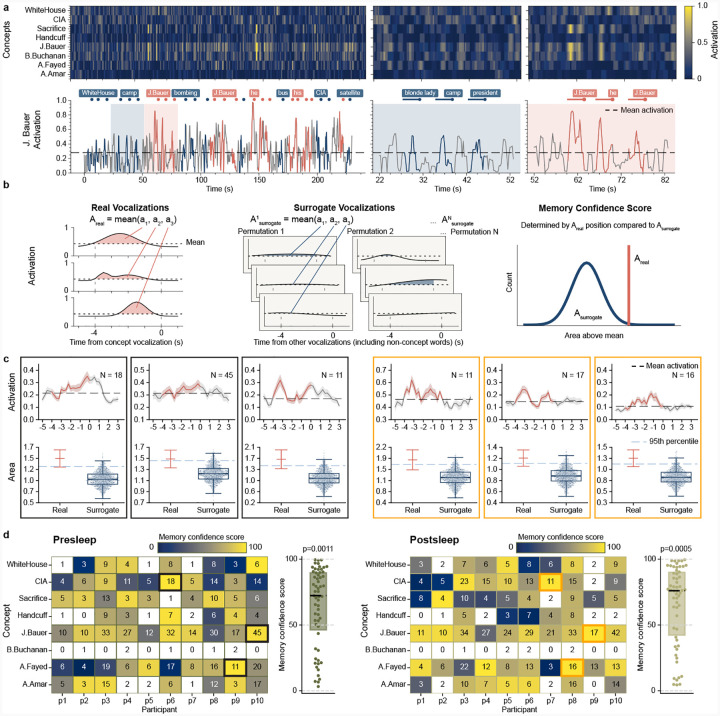
Memory confidence score (MCS) unveils decoding of memories prior to vocalization. **a**, An example model output from a single participant illustrates the MCS procedure. The top-left heatmap shows model activations for all concepts during a 230-second presleep free recall session. Below, a line plot traces the activation of the concept ‘J. Bauer’ over time. Red dots show vocalizations referring to the main character ‘J. Bauer’, blue dots show vocalizations to other concepts, and colored line segments represent 4-second intervals preceding each vocalization onset. The panels in the middle and right figures, zoomed in views of the blue and red shaded sections in the left figure, show stronger activation patterns prior to ‘J. Bauer’ vocalizations compared to unrelated vocalizations. **b**, To quantify the degree of activations prior to related concepts, we compute the area under the activation curve above the mean baseline within each 4-second pre-vocalization window. In the toy example, for a concept mentioned three times (left), the average of these areas defines *A*_real_. We then randomly sample three unrelated vocalizations, compute the same metric, and repeat this process *N* = 1500 times to generate a surrogate distribution $A_{\text{surrogate}}=\{A_{\text{surrogate}}^{\left(1\right)},\ldots ,A_{\text{surrogate}}^{\left(1500\right)}\}$ (middle). We define the MCS as the percentile rank of *A*_real_ within this surrogate distribution. **c**, Six example concepts with high MCS, three from presleep recall (left) and three from postsleep recall (right). Each pair of plots corresponds to one concept. The top plot displays the mean model activation (±SE) aligned to vocalization onset across all occurrences, with the red segment indicating the 4-second pre-vocalization window used for evaluation. The bottom plot visualizes the percentile ranking of *A*_real_ within the surrogate distribution *A*_surrogate_. Real denotes the mean and SE of the activation areas contributing to *A*_real_, while Surrogate shows the distribution *A*_surrogate_ as a swarm plot. d, Heatmaps of population-level MCS across all participants and concepts for presleep (left) and postsleep (right) recall. Each colored cell in the heatmap represents a concept (row) mentioned ≥3x by a participant (column); numbers indicate the total number of vocalizations for that concept by that participant. Concept decoding compared to chance (50%) shows significant effects for both conditions (presleep: p = 0.0011, postsleep: p = 0.0005; Wilcoxon signed-rank), indicating consistent decoding of concepts across participants. The three black outlined cells (left) represent the presleep examples in c and the three orange outlined cells (right) represent the postsleep examples in **c**.

**Fig. 3:**
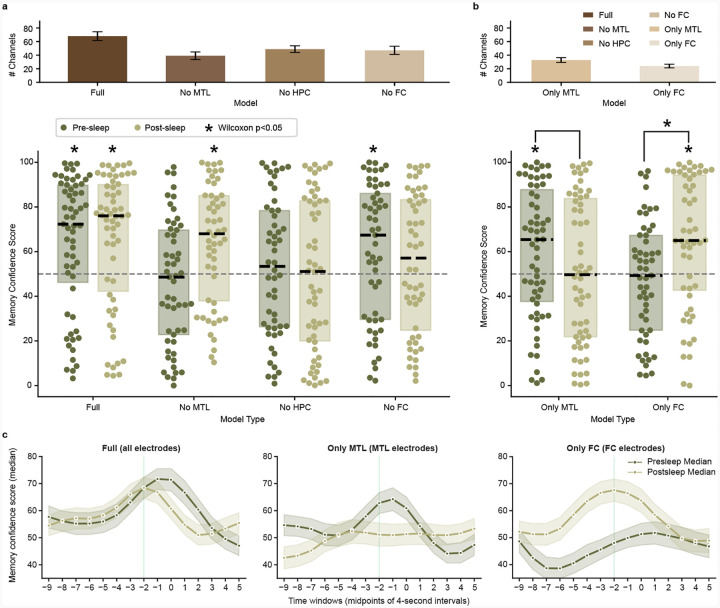
Medial temporal lobe (MTL) and frontal cortex (FC) differentially contribute to pre- and postsleep memory decoding. **a**, **Top**: mean±SE channel counts for each model; **Bottom**: Presleep and postsleep memory confidence scores for the Full model (same data as Fig. 2d) vs. models with removal of clusterless voltage spike data recorded in MTL (including hippocampus), hippocampus (HPC), and FC. Each dot shows a single concept decoded for the given model trained on an individual participant’s data, dashed black lines show median, rectangles show interquartile range, and asterisks indicate significance of a Wilcoxon signed-rank test for concepts grouped across participants vs. chance (50% decoding). Note that the Full and MTL models include all (N=10) participants, while the No HPC (N=9) and No FC (N=9) models test only those participants that originally possess those regions (Table 1). **b**, Same conventions as **a**, but for models restricted to channels localized in MTL (Only MTL, N=10) and FC (Only FC, N=9). **c**, Our main results suggest that internal reinstatement of concepts during recall occurs prior to vocalization. To confirm our models show peak decoding during this putative window of reinstatement, we ran a series of stepwise models using an identical procedure as Fig. 2a–b to calculate MCS at every four-second window centered from −9 to +5 seconds in reference to vocalization times. For example, the −2 s point in these plots inferences on neural data in the window −4 to 0 s from vocalization (all MCS reported prior to this plot have used this −4 to 0 s window, as we expect memory reinstatement of concepts to occur as participants begin to say the sentences containing the concept vocalizations). The Full model (left), Only MTL model (center), and Only FC model (right) all show peak decoding in windows centered 1 to 2 seconds prior to vocalization for both pre- and postsleep.

To evaluate the contribution of specific brain regions on the performance of our memory decoder, we trained several additional models that excluded channels based on electrode localization (Fig. 1a, right and Table 3). These models include removal of MTL (No MTL, which excludes microwires in hippocampus, amygdala, parahippocampal cortex, and entorhinal cortex), hippocampus (No HPC), and FC (No FC, which excludes microwires in orbitofrontal, superiorfrontal, inferiorfrontal, and anterior cingulate cortex). For each model we then measure the MCS both before and after sleep—using the same process shown in Figs. 2 and 3—to assess whether we can decode concepts without the contribution from the removed region.

For the nine participants with microwires localized to both MTL and at least one other region (since a participant without MTL coverage must have remaining channels to run a model), the No MTL model fails to decode concepts above chance level in presleep memory tests (Fig. 3a; *Z* = −0.961, *p* = 0.3368, *D* = −0.139) but successfully decodes them in postsleep (Fig. 3a; *Z* = 3.233, *p* = 0.0012, *D* = 0.511). For the nine participants with microwires localized to both hippocampus and at least one other region, the No HPC model fails to decode concepts above chance level for memory tests both presleep (Fig. 3a; *Z* = 1.249, *p* = 0.2116, *D* = 0.164) and postsleep (Fig. 3a; *Z* = 0.190, *p* = 0.8496, *D* = 0.019). For the nine participants with microwires localized to both FC and at least one other region, however, the No FC model successfully decodes concepts above chance level for memory tests presleep (Fig. 3a; *Z* = 2.324, *p* = 0.0201, *D* = 0.322) but not postsleep (Fig. 3a; *Z* = 1.248, *p* = 0.2121, *D* = 0.164). These results suggest MTL—particularly hippocampus—provides crucial inputs to the full model both before and after sleep, while FC contributes during postsleep.

Considering that removal of MTL and FC channels impact our full model in different ways, we ask if models trained using only MTL (Only MTL) or only FC (Only FC) can decode concepts during recall. Table 3 summarizes the number of microwires from each region, with all 10 participants possessing at least 16 microwires in MTL and nine participants possessing at least 16 microwires in FC. We find that the Only MTL model successfully decodes concepts above chance level for memory tests presleep (Fig. 3b; *Z* = 2.359, *p* = 0.0183, *D* = 0.321) but not postsleep (Fig. 3b; *Z* = 0.337, *p* = 0.7363, *D* = 0.030) albeit with no significant difference when comparing the MCS distributions directly (*Z* = 1.120, *p* = 0.2695, *D* = 0.181; paired Wilcoxon test and Cohen’s D for paired samples for all paired tests). The Only FC model, meanwhile, fails to significantly decode concepts above chance level during presleep memory tests (Fig. 3b; *Z* = −0.518, *p* = 0.6045, *D* = −0.089) but does significantly decode them during postsleep (Fig. 3b; *Z* = 3.033, *p* = 0.0024, *D* = 0.470), with a significant difference when comparing them directly (*Z* = 2.060, *p* = 0.0391, *D* = 0.332). Combined, the Only MTL and Only FC models represent a double dissociation between pre- and postsleep decoding, with Only MTL capable of decoding concepts before sleep and Only FC capable of decoding concepts after sleep, but not vice versa.

## Decoding not related to cell selectivity

**Fig. 4:**
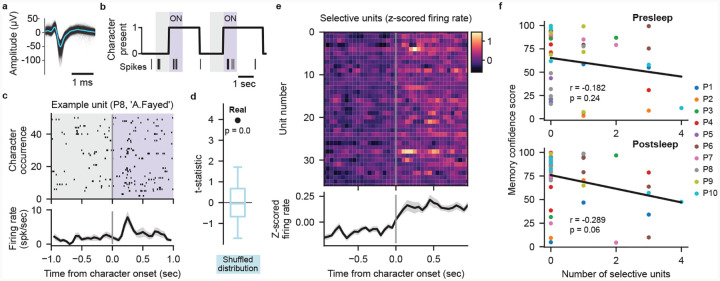
Character-selective cells cannot explain decoding performance. **a**, Waveforms of a manually spike sorted single unit (black lines) overlaid with the mean waveform (blue line). **b**, Example labeling for presence of character ‘A.Fayed’, with spiking activity of an example character-selective unit (unit shown in (**a**)) shown as vertical ticks below. Shaded regions indicate analysis windows 1 second prior to character onset (gray) and 1 second post-onset (purple). **c**, Spiking activity and peristimulus time histogram (PSTH) of the example unit in (**a**) over 51 appearances of ‘A.Fayed’. PSTH (bottom) black line shows mean firing rate across appearances in 100 ms bins with 50 ms overlap, gray shade represents SE. **d**, Observed t-statistic (‘Real’) for the unit in (**a**) with the permutationgenerated surrogate distribution of t-statistics (blue; box plot indicating median, interquartile range, and 5th and 95th percentiles) used to determine the significance of firing rate increase. **e**, Firing rate PSTHs of individual character-selective units (*p* < 0.01, permutation test) with mean (black line) and SE (gray shading) across all selective units. **f**, Relationship between the Memory Confidence Score (MCS) and the number of selective units for each concept. Each dot represents a single concept decoded on an individual participant’s data. No positive correlation is observed between the number of selective units and MCS for both presleep (Pearson correlation, *r* = −0.182, *p* = 0.24) and postsleep (*r* = −0.289, *p* = 0.06) recall.

Does the extraction of specific memories achieved by our model depend on the recruitment of cells specifically responsive to concepts comprising those memories? In the human MTL, concept cells frequently respond to characters in visual media[19, 20]. We therefore investigate to what degree neural firing from potential concept cells evoked by the presence of characters in the video contributes to our transformer decoding performance. We surmise that if character-selective units provide the primary source of information for our models then we should see a positive correlation between the number of selective units and the corresponding MCS for each concept. Therefore, we generated frame-level labeling of on-screen character presence for 10 main characters that map onto our decoded concepts (Methods). Taking 626 manually sorted single- and multi-units combined from all 10 participants (Fig. 4a, Supplementary table 3), we use a permutation t-test to identify units that significantly increase in firing rate when a given character appears onscreen (Fig. 4b–c; Methods). We find 37 units that selectively respond (*p* < 0.01 permutation t-test) to a single character across our participants (Fig. 4e; Extended Data Fig. 4a). Correlating the number of selective units for each concept within each participant to the corresponding MCS reveals no positive relationship between them for either pre- or postsleep recall, with both correlations trending negative (Fig. 4f). Including units that respond to more than one character, we again find no positive correlation between the number of responsive units and MCS (Extended Data Fig. 4b–d). We also find no significant difference between the MCS values for concepts with or without selective units for both presleep (concepts with selective cells *n* = 18, median MCS = 65.10; concepts with no selective units *n* = 26, median MCS = 75.60; Wilcoxon ranksum test *Z* = −1.34, *p* = 0.18) and postsleep (concepts with selective cells *n* = 17, median MCS = 70.50; concepts with no selective cells *n* = 27, median MCS = 82.40; Wilcoxon ranksum test *Z* = −0.57, *p* = 0.57) recall. These results suggest that character-responsive units do not centrally contribute to how our model decodes concepts during recall.

## Discussion

We asked if small populations of neuronal spikes recorded within individual participants could uncover recalled memories using recordings taken during initial memory formation. While previous work has shown successful classification of on-screen characters using part of the viewing data for training and the other part to perform inference[12–14], we report a fundamentally different achievement: predicting concept identity from neuronal patterns during out-of-sample, internally-generated reinstatement. Our deep learning method, trained on a single viewing of an audiovisual episode, successfully predicts subsequent recall of concepts using recordings from only 40–96 sites (channels) across the brain, mostly in medial temporal lobe (MTL) and frontal cortex (FC). As clinical considerations solely determine the location of recording sites, our models achieve decoding from a relatively small, nearly arbitrary complement of microelectrodes without curating brain regions or neurons with response characteristics. When we restrict our model inputs to only MTL or FC spikes, we successfully decode concepts in presleep or postsleep, respectively. Our models do not appear to take advantage of traditional single neuron concept selectivity[19], however, suggesting nonlinear interactions at the level of population neuronal spikes contain information useful to decoding of internal brain representations.

The notion that memory retrieval involves reinstatement or reactivation of the neural state active during encoding represents a central and well-supported theory in cognitive neuroscience[35], although showing reinstatement of a specific memory in a single recollection remains challenging. At the cellular level, studies using place cells in rodents demonstrate reinstatement before, during, and after sleep[36]. In humans, reactivation of MTL concepts cells was shown just prior to memory recall of their selected concepts[20] and neuronal sequences in lateral temporal cortex that occur immediately after encoding of paired words subsequently replay prior to correct cued recall[37]. These methods require identification of concept neurons or characteristic neural sequences to uncover internal representations, and in the latter case these neuronal sequences appear to possess a general role in representing categorical information[38]. In contrast, our work represents decoding of conceptual information during memory retrieval at the level of population neuronal activity without identifying neurons or sequences responsive to stimulus viewing, likely relying on dynamic interactions distributed widely over frontotemporal regions.

Systems consolidation theory posits that new memory formation depends on the hippocampus before reorganization into hippocampally-independent traces[39]. Sleep represents a critical period for this consolidation process[27]. Our brain region–specific modeling results suggest systems consolidation can be seen at the level of population neuronal spikes (Fig. 3), as demonstrated by a striking difference between decoding of memories before sleep and after sleep. Before sleep, our “Full” model successfully decodes concept recall, while models without MTL, or without hippocampus, fail to do so. Concurrently, the “Only MTL” model, which excludes FC sites, can significantly decode concepts before sleep while the “Only FC” model does not. Following sleep, the “Full” model can still decode concept memories, as can the “Only FC” model, but the “Only MTL” fails. Our presleep findings are compatible with work showing reinstatement of specific memories via activity of single entorhinal-hippocampal concept neurons prior to recall vocalizations before sleep, while frontal cortex neurons with selective responses to concepts fail to do so[20]. Neurons in that study, however, were not followed to postsleep recall. Our study demonstrates reinstatement of FC activity dominates postsleep decoding, suggesting that specific information in FC neurons during encoding reinstates during memory recall after sleep.

The present study supports the transformation of memory representations from MTL to neocortex, highlighting the dynamic nature of the representation as long-term memories take hold. MTL and FC play central roles in memory[40] with human neuronal interareal communication between them strengthening during successful memory retrieval[41] particularly during periods of high cognitive demand[17]. Evidence suggests MTL memory independence can occur over the course of a single night of sleep[42, 43], supporting the rapid shift from MTL to FC–dependent decoding before and after sleep. Relatedly, we find FC contributes to postsleep decoding after failing to contribute to presleep decoding (Fig. 3b). This reemergence of previously silent, memory-related neural patterns in postsleep FC may reflect activity silence [23, 44] as MTL dominates presleep reinstatement[5]. An alternative explanation could be slow learning in FC that only matches the neural patterns from viewing after overnight consolidation into existing schema[45].

Our decoding model shows little relationship between the presence of character-selective cells and memory decoding, with no significant difference between MCS when a given concept has selective units (≥1 selective unit) or none (0 selective units). These results dovetail with our inability to decode concepts with logistic regression (Extended Data Fig. 2), as this model relies solely on tuning of neuronal spikes to concept presence during episode viewing to predict retrieval of the concept during subsequent recall. Previous results have shown that picture identity can be decoded from selective MTL neurons, but required a curated population of spike sorted neurons (19 neurons known to respond to at least one of 32 images[16]). Considering that our selective cell numbers (37*/*626 ≈ 6%) match previous results showing only ~ 4% of MTL neurons respond to a set of common objects[23]), our results suggest the low neuron yield of current recording methods constrains concept memory decoding from concept-selective firing rates, particularly for a new experience. Confirming this limitation, recent results suggest character responsive neurons contribute little to the success of LSTM models capable of decoding character identity from intracranial neural data recorded as participants watched a TV episode[13].

In sum, our results suggest deep learning models take advantage of patterns in the neural code beyond visual tuning, which could include spike timing and nonlinear interactions between groups of neurons[33]. As transformer models have proven successful in part due to their attention mechanism, which enables, for example, language and vision decoders to capture long-range dependencies between distant words or pixels[2, 3], it is plausible that coincident neural spikes across multiple channels and time points similarly contribute to our decoding success.

Accumulating evidence suggests that deep brain stimulation can improve memory, where electrical stimulation of the entorhinal-hippocampal system[46, 47] or neocortex during awake encoding[34] or sleep consolidation[48] can enhance memory retrieval. Modulating a specific human memory, however, has remained a formidable challenge. Since our models provide a framework to decode specific memory content within individuals, future work timing stimulation to successfully decoded concepts has the potential to selectively enhance memory for that content. Previous models that decode internal representations of videos relied on separating training and test data from the same viewing session[12, 13], making it difficult to separate perceptual responses from internally-generated concepts. Promising fMRI work has shown the ability to decode the gist of semantic content[49], but required several hours of training and remains impractical for device applications. Our ability to train single-participant models on single-shot, unsorted neural recordings enables inference of conceptual memory content in real time. Inference in deep learning-based architectures can be executed within tens of milliseconds on standard hardware, as demonstrated in real-time neuroprosthetic systems [31, 32], and is compatible with closed-loop stimulation applications. Methods to predict specific memory content can therefore serve as a basis for human memory neuroprosthetic devices aimed at modulating specific memories.

## Methods

### Participants

Ten particpants with drug-resistant epilepsy (2 females, age range 24–55 years, average age 36.9±3.9 years) who were implanted with intracranial depth electrodes for seizure monitoring participated in our study. The study protocol was approved by the Institutional Review Board (IRB) of UCLA and The University of Iowa and all participants provided written informed consent prior to participation.

### Behavioral tasks

Participants viewed a 42-minute episode of 24 (Season 6, Episode 1), followed by memory tests conducted before and after an overnight sleep session (Fig. 1a). The memory tests typically comprised of three components: free recall, recognition memory, and cued recall (P1 and P2 only performed free, not cued, recall). In free recall, participants were asked to verbally recall all details they could remember about the episode. In the recognition memory task participants were shown 150 short clips (0.5 – 5 seconds; 75 target clips from the episode they watched, 75 foil clips from another episode in the season) in a random order and were tasked to respond whether the clip was from the episode they had watched or not. In cued recall, participants verbally answered six image-based questions. Recall audio was recorded at 32 kHz using the Neuralynx ATLAS system. A combination of automated methods and manual curation were used to annotate microphone recordings of verbal recall to timestamp word vocalizations.

### Neural recordings

Participants were implanted with Behnke-Fried hybrid depth electrodes (AdTech Medical) with placement determined solely by clinical criteria. Electrophysiological data were recorded using the Neuralynx ATLAS system. Intracranial EEG (iEEG; 0.1 – 500 Hz; 2 kHz sample rate) signals were recorded from macroelectrode contacts (1.5 mm wide) spaced along the probe shaft. Broadband potentials (0.1 Hz - 8 kHz; 32 kHz sample rate) were recorded from bundles of nine 40 *μ*m Platinum-Iridium microwires (8 high-impedance recording electrodes and 1 low-impedance reference wire) that were threaded through the depth electrodes.

### Electrode localization

We localized electrodes to brain regions by loading a pre-operative T1-weighted MRI (1mm isotropic) and post-operative high-resolution CT (<0.6mm × <0.6mm × 3mm) into Locate electrodes Graphical User Interface (LeGUI) software[50]. In LeGUI, the MRI and CT images were co-registered, normalized to the Montreal Neurological Institute (MNI) template, macroelectrode contacts were automatically segmented, and microelectrode contacts were manually identified from the CT scan by an expert rater. Quality control for all contact localizations was done by visual inspection during contact labeling. We segmented cortical and subcortical regions automatically using FreeSurfer [51]. Medial temporal lobe contacts, which cortical parcellation software can fail to properly identify, were automatically segmented using Automatic Segmentation of Hippocampal Subfields (ASHS) software with the ASHS ABC 3T protocol[52, 53]. Electrode contacts within the ASHS segmentations were manually labeled by visual inspection. Coordinate transformations from LeGUI to ASHS and FreeSurfer spaces was performed using FSL software[54].

### Model input data generation

#### Voltage spikes.

Broadband microwire signals were notch filtered (4th order Butterworth, center frequencies from 300 to 3000 Hz in steps of 60 Hz, bandwidth 4 Hz) to reduce line noise, and high-pass filtered (4th order Butterworth, cutoff at 300 Hz) with zero-phase lag filters to isolate neural spiking events. To detect voltage spike events, we first computed the standard deviation (SD) of the filtered signal amplitude separately for each channel and task epoch (e.g., movie viewing, recall, sleep). The minimum SD across epochs for each channel was used to set a symmetric threshold at ±3×SD, which was then applied across all task epochs for that channel. We identified threshold-crossing events in both directions: negative-going deflections and positive-going deflections. Each threshold-crossing was considered a putative voltage spike. For negative crossings, we recorded the minimum amplitude and its corresponding time point. For positive crossings, we recorded the maximum amplitude and its time point. These values were treated as separate events to preserve the bidirectional structure of extracellular voltage signals. The resulting time series of amplitudes, one for negative and one for positive events, constituted the voltage spike amplitude trains. To reduce artifacts, we removed coincident events across channels within a microwire bundle. Specifically, if synchronous events were detected across 6 of 8 channels within a 4-ms window, we excluded these values from both the negative and positive amplitude trains. To facilitate comparability across channels and participants while preserving the relative structure of neural firing patterns, we discretized spike amplitudes into fixed bins defined by multiples of the detection threshold’s standard deviation (SD). The amplitudes were grouped into half-width intervals ranging from 3.5 to 30.5 (e.g. [3.5, 4), [4, 4.5), … , [30, 30.5)), and each value was replaced by the left boundary of its corresponding bin. This discretization serves to reduce sensitivity to minor amplitude fluctuations while retaining key information about relative spike intensity. Voltage spike trains were segmented into 250-ms intervals aligned with the movie episode. After excluding the end credits, the episode spanned 2,435 seconds, yielding 9,740 windowed neural segments. Each 250 ms neural signal from a single electrode was initially represented as a 2D array of shape 2 × *N*, where *N* = *f*_*s*_ × 250*ms* = 8,000, and the two rows encode the discretized amplitude values from negative- and positive-going threshold-crossing events, respectively. To reduce dimensionality while preserving temporal structure, we applied a non-overlapping 5-ms binning window independently to each polarity channel. Each bin contained *L* = *f*_*s*_ ×5*ms* = 160 samples, resulting in a total of *B* = *N/L* = 50 bins per segment. Within each bin *j*, we sum the discretized amplitude values. The final processed data for each 250-ms window were structured into a tensor $w_{i}\in {\mathbb{R}}^{2\times N_{e}\times B}$, where *N*_*e*_ is the number of electrodes, *B* = 50 is the number of temporal bins, and the first dimension indexes spike polarity, distinguishing negative- and positive-going deflections. We normalized the binned amplitudes by z-scoring them within each recording bundle, using the mean and standard deviation calculated over the entire experimental period. The normalization was performed separately for negative- and positive-polarity channels.

#### Concept labels.

We manually annotated each second of the episode, identifying concepts of interest displayed on the screen. Each second interval was assigned a vector as its label, where the length of the vector corresponds to the total number of concepts. Each index in the vector represents a specific concept, with 1 indicating the concept’s presence and 0 indicating its absence. This format allows multiple concepts to be assigned to the same time interval, reflecting the possibility of co-occurring concepts. To ensure sufficient training data, we divided each second into four equal parts, resulting in 250 ms annotation intervals, where each quarter-second shared the same label. The identified concepts span three themes: characters, locations, and abstract concepts. Their temporal presence is visualized in Fig. 1c (bottom). The concepts of interest were selected based on their frequency of appearance during the episode and their frequency of mention during the recall of the participants. Eight key concepts were identified: “White House” (includes “Washington”, “DC”, “President”, “D. Palmer” [the President’s name]), CIA (includes “FBI”, “DHS”, “CTU” [the actual name of the government organization in the show, which is typically confused for CIA], or “Chloe” [main character in CTU scenes]), “sacrifice” (includes “hostage”, “exchange”, or “trade”), “handcuff” (includes “ties”, “bindings”, “restraints”, or “chains”), J.Bauer (includes “main character” and any reference to the main agent), B.Buchanan, A.Fayed (includes “head of terrorist organization” and any reference to main bad guy setting the trap), and A.Amar (includes neighborhood “son” or “kid” in reference to his father being detained). For vocalizations of the same concept within 5 s of each other, we removed all but the first mention to avoid overlapping model predictions in our primary −4 to 0 s window. We included pronouns that refer to these concepts in our annotations when the subject referred to by the participant is clear, as we expect them to evoke similar neural reinstatement as nouns[55]. Note that translation of vocalizations and accumulation into the eight categories was always finished prior to any model training and never changed to avoid experimenter influence. To account for temporary absences of concepts due to camera movements, scene cuts, or transitions, we filled the gaps in the labels for each concept (e.g., as shown in Fig. 1c (bottom), J. Bauer was considered onscreen as long as he was present in a given scene). We hypothesized that these brief absences would not affect the viewer’s perception of the continuous existence of the concept. This adjustment ensures that the labels better reflect the viewer’s likely interpretation of the episode. Labels were aligned with the neural data for each participant using audio alignment of the episode audio track to microphone recordings taken during the experiments.

### Model architecture

Our model implementations were carried out using the Python and PyTorch frameworks. The model architecture comprises four key components: (1) a set of region embedding layers that transform neural signals from each microwire bundle into feature matrices while applying positional encodings to retain temporal information, (2) a multiregion encoder that includes region-wise self-attention layers to model intraregion relationships and cross-regional attention layers to capture interactions between different bundles, (3) a combiner module that aggregates the outputs from the multiregion encoder to produce a unified representation of collective neural activity for each 250-millisecond interval (50 time bins), and (4) a classification head comprising a single linear layer that maps the unified representation to the probabilities of the eight main concepts.

**Extended Data Fig. 1:**
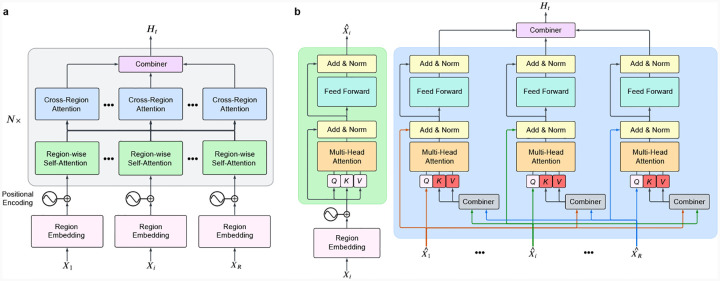
Overview of the multi-region transformer architecture. **a**, We process neural signals (*X*_1_, *…,X*_*i*_, *…,X*_*R*_) from distinct brain regions through region-specific embedding layers, followed by region-wise self-attention and cross-region attention layers, and finally integrate by a combiner module to produce a unified representation of neural activity *H*_*t*_. The classification head predicts the probabilities of eight target concepts for each 250-ms interval. **b**, Detailed structure of the Region-wise Self-Attention (RSA) and Cross-Region Attention (CRA) layers. The RSA layer captures intraregion dependencies by attending to relationships within the same region, including interactions with the class token. The CRA layer processes outputs from RSA layers, enabling interactions across brain regions by aggregating information from all class tokens. Arrows illustrate the data flow within and across regions.

An overview of our transformer architecture is shown in Extended Data Fig. 1a. Neural signals were partitioned by microwire bundle, with each region contributing 8 electrodes to the model input. We denote the neural activity from the *R* brain regions as **x** = (*X*_1_, *…,X*_*i*_, *…,X*_*R*_), where each *X*_*i*_ represents the neural signal from region *i*. The dimensions of *X*_*i*_ vary depending on the data type. For clusterless data, each *X*_*i*_ is represented as a tensor of shape (2, $N_{e}^{i}$, *B*), where $N_{e}^{i}$ is the subset of electrodes corresponding to region *i*, and *B* is the number of time bins within the 250-ms interval. The total number of electrodes across all regions satisfies $\sum_{i=1}^{R}N_{e}^{i}=N_{e}$. Similarly, for macro iEEG data, each *X*_*i*_ has dimensions (1, *H*_*i*_, *T*), where *H*_*i*_ is the subset of electrodes assigned to region *i*, each decomposed into eight frequency bands, and *T* represents the temporal dimension sampled within the 250-ms window. The total number of frequency-augmented electrodes satisfies $\sum_{i=1}^{R}H_{i}=H$.

Each region-specific input *X*_*i*_ is first processed by the region embedding layer, where it is transformed into a feature embedding augmented with positional encodings to preserve temporal information. These embeddings are processed by the multiregion encoder, which consists of two sequential mechanisms: region-wise self-attention (RSA) for intra-region dependencies and cross-region attention (CRA) for inter-region interactions. Inspired by [4], this design enables effective integration of information across regions through structured attention mechanisms. The encoded outputs are then aggregated by the combiner module, producing a unified representation of neural activity. Finally, a classification head, implemented as a single linear layer, predicts the probability that a concept is active within the given 250-ms interval.

#### Region embedding.

The region embedding layer follows the patch embedding approach used in Vision Transformers (ViT) [3]. Each neural activity matrix *X*_*i*_ is processed using a convolutional neural network (CNN), partitioning it into non-overlapping patches that serve as local feature representations. The convolution operation preserves spatial relationships while reducing dimensionality. A class token, a learnable vector widely used in ViT, is concatenated with the patch embeddings to provide a global representation of the entire region. During attention operations, this class token aggregates information from all patches, allowing the model to capture region-wide neural dynamics. The processed embeddings are then passed through positional embeddings and subsequently to the transformer layers.

#### Multi-region encoder.

The multi-region encoder models both local (within-region) and distributed (cross-region) neural interactions, incorporating region-wise self-attention (RSA) and cross-region attention (CRA). The RSA layer captures intraregion dependencies, refining neural activity within each region, while the CRA layer enables inter-region interactions by integrating information across different brain regions.

#### Region-Wise Self-Attention (RSA).

The RSA layer captures interactions within the same brain region by applying self-attention to the region embeddings. Each region embedding is processed using self-attention, where queries, keys, and values are computed from the same feature matrix. The attention mechanism dynamically prioritizes relevant spatial and temporal features, refining the representation of the neural activity within each region. At the output of RSA, only the updated class token, denoted as ${\bar{X}}_{i}$, is retained, while the patch-level representations are discarded. This compressed representation of region *i* is then passed to the cross-region attention (CRA) layer, ensuring that inter-region interactions operate on compact yet informative summaries rather than the full feature maps (Extended Data Fig. 1b).

#### Cross-Region Attention (CRA).

The CRA layer models inter-region dependencies by processing the class tokens output from RSA. Unlike RSA, where attention operates within a single region, CRA computes queries from the class token of a given region, while keys and values are derived from the class tokens of all other regions. Specifically, for region *i*, the input to CRA is the updated class token ${\bar{X}}_{i}$, while the set of keys and values is constructed by concatenating class tokens from all other regions, $\left\{{\bar{X}}_{j}|j\neq i\right\}$. This allows the model to selectively integrate inforamtion from other brain regions, captureing inter-regional interactions (Extended Data Fig. 1b). The output of CRA is an updated representation of ${\bar{X}}_{i}$, now enriched with context from the other regions. This refined set of class tokens is then passed to the combiner module, where all regional embeddings are aggregated into a unified latent representation of neural activity.

#### Combiner.

The combiner module consolidates the outputs of the cross-region attention layers to generate a final unified representation of neural activity. Prior work [4] explored both weighted summation and unweighted averaging strategies for this aggregation, reporting similar performance between the two approaches. Based on these findings, we adopt the simpler approach of averaging the regional representations to produce the final latent representation *H*_*t*_.

#### Further training details.

We expect the overall brain state and associated neuronal activity patterns during recall of one-time viewed episodes to differ substantially from those during viewing, as recall relies on internally generated signals without concurrent sensory input. When participants view the episode, we expect two distributions of neural patterns: those responsible for perceiving the rich, continuous stream of visual and audio content and those that form memories[40]. During recall, external stimuli from the episode no longer exist, meaning the relevant reinstatement of concepts may only rescue a small component of the original audiovisual percepts[56, 57]. This difference is amplified in postsleep recall due to potential neural reorganization or electrode drift. This leads to a machine learning scenario in which training and testing data are drawn from different distributions, with only a limited shared component. Although prior work has proposed methods to address such distributional shifts through domain adaptation and neural manifold alignment[58, 59], these approaches often require densely labeled or trial-based data, which is impractical in our freerecall setting with sparse and unconstrained concept occurrences. We therefore adopt a strategy of learning the training signal as faithfully as possible and use the entire viewing period for training the model to reduce training error. During recall we expect the trained models to respond to only one of the relevant components in the neuronal activity mixture, potentially leading to an output below 0.5 even for a successfully decoded concept. This motivated our use of the Memory Confidence Score (MCS; detailed below), which compares prediction statistics before concept recall to those from matched surrogate intervals.

The model was trained using binary cross-entropy (BCE) loss for a training duration of 49 epochs with a learning rate of 1e-4, optimized using Adam and scheduled using StepLR. The transformer architecture consisted of a hidden size (feature embedding length) of 396, an intermediate (feedforward) size of 792, model depth of six RSA and CRA layers, and six attention heads (Extended Data Fig. 1). We tuned hyperparameters based on empirical performance across four participants (P3, P6, P9, P10) who exhibited rich verbal narratives during both pre- and post-sleep recall. These hyperparameters included variations in model depth, hidden size, and training duration, and we selected the configuration that yielded the highest memory confidence scores across these individuals. Final analysis was then performed on all participants using this set of parameters tuned to the initial four participants. Region embeddings were extracted using CNNs with patch sizes of (1,5) for clusterless data and (1,10) for macro iEEG. To address class imbalance, we performed stratified sampling at the level of unique label combinations, ensuring proportional representation of each distinct co-occurrence pattern among the eight target concepts during training. Combinations with less than 50 seconds of total screen time were excluded, along with their corresponding neural data segments. Rather than applying cross-validation or creating held-out sets from the movie viewing data, we trained in a fully supervised manner using all available labels, prioritizing generalization to the recall phase.

### Model inference on recall data

We applied our trained models to each participants’ recall data, combining free and cued recall where both were available (for P1 and P2 only free recall was available). We used the same preprocessing and voltage spike extraction methods that were applied to the training data on the microwire signals recorded during recall. As above, we partitioned data into 250 ms bins on which the model performed inference. The model output an activation between 0 and 1 for each concept independently over time (i.e. activation across concepts was not required to sum to one). The activation values indicate how strongly the neural patterns at each moment of the recall data matches the signature for the concepts that the model learned during training.

### Memory confidence score

To assess decoder performance during recall, we first smoothed the time-resolved model activation for each concept using a moving average with a 1-second window. We then evaluated these smoothed activations for each concept relative to vocalization onset of that concept. For each instance in which a participant vocalized a concept, we extracted the activation signal within a 4-second window preceding the vocalization. We computed the area under the activation curve exceeding a baseline (the mean activation level for that concept across the entire recall period). The average of the areas above mean across all vocalizations of the concept was denoted as the observed activation score, *A*_*real*_ (Fig. 2b, left, shaded region). To evaluate significance, we performed a permutation test. For each concept, we randomly sampled the same number of 4-second windows from time points preceding unrelated words (excluding true vocalizations of the given concept, including other concept vocalizations), computed the area under the activation curve exceeding the mean activation level, and averaged them to obtain a surrogate activation score, *A*_*surrogate*_. This procedure was repeated 1,500 times to generate a null distribution ${\left\{A_{\text{surrogate}}^{\left(k\right)}\right\}}_{k=1}^{1500}$ (Fig. 2b, middle). The memory confidence score (MCS) was defined as the percentile rank of *A*_*real*_ within this distribution. Higher scores reflect more reliable concept-specific activations.

### Logistic regression

To run logistic regression models, we replaced the RSA and CRA layers in the transformer architecture with a single linear layer and flattened the input into a onedimensional feature vector, which directly mapped to concept labels. All other training settings were identical to those used for the transformer models.

### Macrowire iEEG trained model

Macro iEEG data were bipolar referenced before spectral decomposition using a Morlet wavelet transform, extracting the instantaneous powers of eight logarithmically spaced frequency bands ranging from 3 to 180 Hz. As with the microwire model input, each training data point was represented as a tensor $w_{i}^{\prime }\in {\mathbb{R}}^{1\times H\times T}$. Here, $H=N_{e}^{\prime }*8$ corresponds to the appended number of re-referenced electrode pairs $N_{e}^{\prime }$, each decomposed into eight frequency bands, and *T* corresponds to the temporal dimension derived from the 250-ms window sampled at $f_{s}^{\prime }$. The model architecture, training procedure, and MCS evaluation metrics remained as described above.

### Circular shift permutation test

To evaluate whether the decoder performance reflected meaningful structure in the data rather than random associations, we performed a circular shift permutation test (n=100 permutations) that disrupts label alignment while preserving the temporal continuity of both the neural input data and movie label data. Specifically, we displaced the neural data along the time axis by the same magnitude across all channels, with any data that extended beyond the end of the time axis wrapping back to the beginning. Within a given permutation the neural data for each participant was shifted by the same magnitude. This preserves the internal temporal structure within each 250-ms bin, as well as the vast majority of the structure across bins, but breaks the alignment with the movie-derived labels. This method serves as a conservative control, particularly given the structured nature of the movie stimulus, which includes correlated and repeating events. For each of the shifted datasets we trained transformer models for each participant, performed inference on the unaltered recall data, and calculated a Memory Confidence Score (MCS) for each concept using the same procedure as described above. We performed a Wilcoxon signed-rank test against 50% for the pooled MCSs across participants in each permutation to generate a surrogate distribution of 100 Z-statistics for presleep recall and 100 Z-statistics for postsleep recall.

### Character selective units

#### Spike sorting and unit analysis.

We used WaveClus3[60] for automated spike detection and sorting. We then manually curated the automated sorting output by evaluating the waveform shape, inter-spike interval violations, and firing consistency across recording sessions. Units with a firing rate across the video viewing period of < 0.5 spk/sec were excluded from further analysis. Z-scored firing rates were calculated using the mean and standard deviation of the binned firing rate (100 ms bins) across the full episode.

#### Character labeling and mapping to concepts.

We used custom software to annotate the on-screen presence of 10 characters in the 24 episode at frame-level resolution (29.97 frames per second). Character presence was determined solely by their visual appearance in each frame. The 10 characters we labeled were unique to the concepts used for our decoding. Some decoded concepts mapped directly to characters (A.Amar, A.Fayed, J.Bauer) whereas for other concepts we assigned multiple characters (K.Hayes, T.Lennox, W.Palmer for ‘WhiteHouse’; C.OBrian, N.Yassir, M.OBrian, M.Pressman for ‘CIA’).

#### Character selectivity analysis.

For a character appearance to be included in this analysis we required that the character appeared alone onscreen for at least 1 second. For each unit we performed a paired t-test comparing the firing rate in a 1 second preonset window (−1 s to 0 s relative to character onset) to 1 second post-onset (0 s to 1 s) for all appearances of a given character. We then compared the observed t-statistic to a surrogate distribution of t-statistics generated by randomly flipping the sign of the pre-to-post onset differences of each individual appearance (n = 1000 permutations). A unit was deemed selective to a character (1) the unit fired at least 1 spike in more than 30% of character appearance post-onset windows, (2) the observed t-statistic exceeded at least 99% of the permuted distribution (*p* < 0.01, one-tailed), and (3) the unit only significantly responded to one character. Running the above analysis with a permutation significance threshold of *p* < 0.05 did not significantly alter the results. We also defined ‘Responsive’ units that adhered to constraints (1) and (2) above, but could respond to more than one character (see Extended Data Fig. 4b–d).

## Extended Data

**Extended Data Fig. 2:**
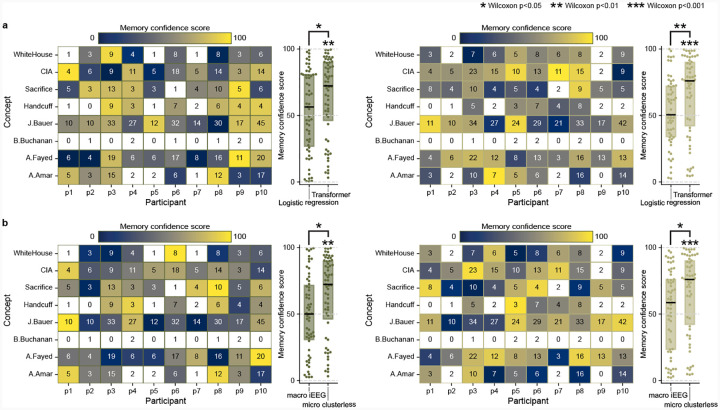
Logistic regression model fails to decode concepts. **a**, For both pre(left) and postsleep (right) memory tests we use a separate multi-class logistic regression model for each participant to decode recall of the same eight concepts and test the performance by calculating MCS as in Fig. 2a–b. Our transformer model performs significantly better than logistic regression for both pre- and postsleep. **b**, For both pre- (left) and postsleep (right) memory tests we run our transformer model on whole-brain, bipolar-referenced macrowire iEEG inputs (sampled at 2kHz) and test them using the same MCS procedure in Fig. 2a–b. These data do not reflect neuron-level spikes in voltages but instead aggregate voltages across hundreds of thousands of neurons for each bipolar channel. Our transformer model with microwire inputs performs significantly better than the model with macrowire inputs for post pre- and postsleep. Statistical tests on individual distributions indicate Wilcoxon signed-rank tests against chance (50%), and tests between paired distributions (shown by brackets) represent Wilcoxon signed-rank tests. *p < 0.05, **p < 0.01, ***p < 0.001.

**Extended Data Fig. 3:**
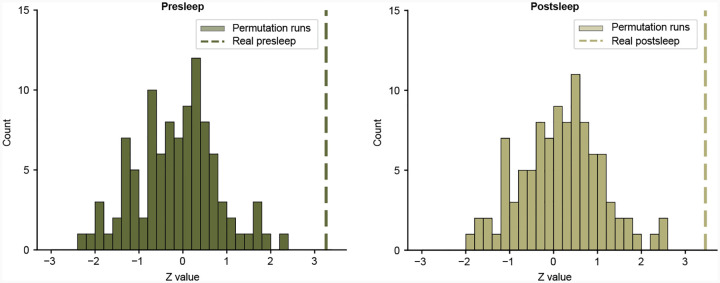
Results of circular shift permutation tests. **a**, Histogram of Z statistics from Wilcoxon signed-rank tests for each of the 100 models with randomly circularly-shifted neural data in reference to the episode annotations (Fig. 1e) for presleep memory tests. We use the same procedure as shown in Fig. 2a–b to calculate MCS and compare to 50% across participants for each model. Dashed line shows the Z statistic for correctly-aligned data *Z*_*real*_ = 3.261 (100th percentile). **b**, Same for postsleep recall. Dashed line shows Z statistic for correctly-aligned data *Z*_*real*_ = 3.457 (100th percentile).

**Extended Data Fig. 4:**
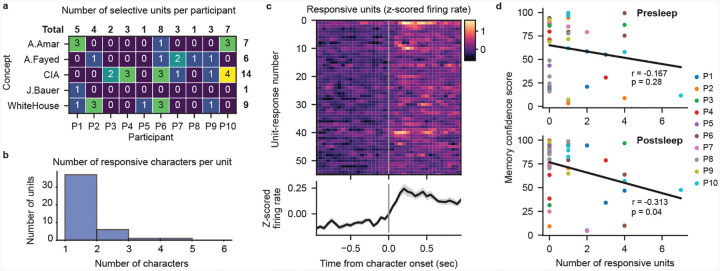
Multi-character–responsive units cannot explain decoding performance. **a**, Significant responsive units by participant for the 37 neurons selective to only one character (*p* < 0.01, permutation t-test). **b**, Eight additional units respond significantly to more than one character. **c**, Firing rate PSTHs of significant character responses, including units with responses to > 1 character (56 character responses over 45 units) (*p* < 0.01, permutation test) with mean (black line) and SE (gray shading) across all units. **d**, Relationship between MCS and the number of responsive units for each concept. Each dot represents a single concept decoded on an individual participant’s data. No correlation is observed between the number of responsive units and MCS for presleep recall (Pearson correlation, *r* = −0.167, *p* = 0.28) and a negative correlation is observed for postsleep recall (*r* = −0.313, *p* = 0.04).

## Supplementary Material

Supplement 1

## Figures and Tables

**Table 1.** Microwire localizations. Each electrode bundle contains eight wires, which we localize by identifying an artifact on the CT scan visible beyond the tip of the macroelectrode sheath. The CT scan is coregistered with participant T1 and T2 structural MRIs to assess the implanted brain region (see further details in Methods). MTL: medial temporal lobe; HPC: hippocampus; AMY: amygdala; PHC: parahippocampal gyrus; ENT: entorhinal cortex; PFC: prefrontal cortex (includes orbitofrontal, superiorfrontal, and inferiorfrontal); ACC: anterior cingulate cortex^1^.

Participant	Microwires per region by participant							
	Full	MTL	HPC	AMY	PHC	ENT	PFC	ACC
P1	48	24	8	0	16	0	8	8
P2	64	32	16	0	8	8	16	8
P3	72	24	16	8	0	0	24	8
P4	40	40	16	8	8	8	0	0
P5	72	32	16	16	0	0	24	16
P6	80	48	16	16	8	8	8	8
P7	72	48	16	16	0	16	8	16
P8	40	16	8	0	8	0	16	0
P9	96	24	0	8	0	16	8	16
P10	96	40	24	8	0	8	24	0

1 FC: frontal cortex as described in the text represents combined PFC and ACC.

**Table 2.** Manually spike sorted unit counts by participant for Fig. 4 analysis.

participant	Total Cell Count
P1	74
P2	49
P3	97
P4	50
P5	78
P6	74
P7	56
P8	30
P9	21
P10	97
